# Development and Validation of a Novel In Vitro Joint Testing System for Reproduction of In Vivo Dynamic Muscle Force

**DOI:** 10.3390/bioengineering10091006

**Published:** 2023-08-25

**Authors:** Yangyang Yang, Yufan Wang, Nan Zheng, Rongshan Cheng, Diyang Zou, Jie Zhao, Tsung-Yuan Tsai

**Affiliations:** 1 School of Biomedical Engineering & Med-X Research Institute, Shanghai Jiao Tong University, Shanghai 200230, China; yang2018@sjtu.edu.cn (Y.Y.); callmebigbig@sjtu.edu.cn (Y.W.); zheng_nan@sjtu.edu.cn (N.Z.); chengrongshan@sjtu.edu.cn (R.C.); zou_diyang@sjtu.edu.cn (D.Z.); 2 Department of Orthopedics, Shanghai Ninth People’s Hospital, Shanghai Jiao Tong University School of Medicine, Shanghai 200011, China; 3 Engineering Research Center for Digital Medicine, Ministry of Education, Shanghai 200230, China; 4 Shanghai Key Laboratory of Orthopaedic Implants & Clinical Translation R&D Center of 3D Printing Technology, Department of Orthopaedic Surgery, Shanghai Ninth People’s Hospital, Shanghai Jiao Tong University School of Medicine, Shanghai 200011, China

**Keywords:** dynamic muscle forces, in vitro biomechanical tests, numerical computation methods, biomedical devices

## Abstract

In vitro biomechanical experiments utilizing cadaveric specimens are one of the most effective methods for rehearsing surgical procedures, testing implants, and guiding postoperative rehabilitation. Applying dynamic physiological muscle force to the specimens is a challenge to reconstructing the environment of bionic mechanics in vivo, which is often ignored in the in vitro experiment. The current work aims to establish a hardware platform and numerical computation methods to reproduce dynamic muscle forces that can be applied to mechanical testing on in vitro specimens. Dynamic muscle loading is simulated through numerical computation, and the inputs of the platform will be derived. Then, the accuracy and robustness of the platform will be evaluated through actual muscle loading tests in vitro. The tests were run on three muscles (gastrocnemius lateralis, the rectus femoris, and the semitendinosus) around the knee joint and the results showed that the platform can accurately reproduce the magnitude of muscle strength (errors range from −6.2% to 1.81%) and changing pattern (goodness-of-fit range coefficient ranges from 0.00 to 0.06) of target muscle forces. The robustness of the platform is mainly manifested in that the platform can still accurately reproduce muscle force after changing the hardware combination. Additionally, the standard deviation of repeated test results is very small (standard ranges of hardware combination 1: 0.34 N~2.79 N vs. hardware combination 2: 0.68 N~2.93 N). Thus, the platform can stably and accurately reproduce muscle forces in vitro, and it has great potential to be applied in the future musculoskeletal loading system.

## 1. Introduction

In vitro biomechanical tests on cadaveric specimens have the unique advantages of being less time-consuming, having controllable experimental variables, and avoiding ethical issues in vivo, among others [1,2,3]. They are widely used to investigate new surgical procedures [4,5], test implants or instruments [6,7,8], and guide rehabilitation exercises [9,10]. Currently, biomechanical testing on specimens is mostly carried out in two main methods, “displacement-controlled” or “load-controlled”. The former generally applies kinematic profiles of given displacements or/and angles to the specimens and then observes their feedback, which is usually the resulting loads. The displacement control method is difficult to apply to biological samples due to the unique kinematics of each individual [11], and a small displacement deviation can generate a large unexpected force on the joint contact surface [3,12,13]. The torque-driven method is one kind of “load-controlled” loading scenario, and it is also the “gold standard” of the current in vitro biomechanical testing [14,15,16]. This method generally applies a pure moment to the synovial or spinal joints as a substitution of the in situ muscle forces simulating in vivo to drive the specimens to generate a passive motion path as a baseline [14,15,17,18]. However, a study demonstrated that the passive motion path differs significantly from the in vivo kinematics in that tibial anteroposterior translation occurs in opposite directions, and the difference for external–internal rotation between in vivo and in vitro ranges from 0° to 21° during a gait cycle [19]. MacWilliams et al. explained that co-contraction of the hamstrings and quadriceps could reduce the internal–external rotation and limits anteroposterior translation [20]. These classical testing approaches, whether “displacement-controlled” or “load-controlled”, ignore the role of skeletal muscles and are restricted to specific input and the researcher’s conception of the situation. They were designed to answer the partial question only [21]. Applying physiological muscle forces on specimens plays a key role in reproducing the in vivo kinematics and bionic mechanical environment.

A few studies attempted to apply muscle forces to in vitro mechanical experiments. Panjabi added an additional set of static muscle forces to the spinal units which were driven by a pure torque and found that the muscle forces increased the range of flexion but decreased the neutral zone [22]. The same method was applied by Li et al. in the in vitro experiment of the knee joint, and they found that muscle forces had different stability effects on different flexion angles [23]. However, static forces cannot simulate muscle loading patterns due to dynamic and diverse changes in muscle forces under functional activities [24,25,26]. For this reason, some studies loaded dynamic forces on the in vitro experimental platform [27]. Ferreira et al. [28] utilized dynamic muscle loading to drive a forearm to complete an active flexion. Recently, Schall et al. [29] tried to add the dynamic muscle forces estimated by inverse dynamics in vivo to the knee joint to complete the jumping movement. However, these studies have some major limitations, such as muscle forces not being a quantitative input, and the measured actual muscle forces being significantly different from the target values. Therefore, proposing a reliable platform based on quantitative inputs for reproducing physiological dynamic muscle forces creates great value.

The objectives of the current study are to (1) develop a platform to reproduce dynamic physiological muscle forces, (2) provide a numerical computation method to reproduce muscle force based on the current platform hardware accurately, and (3) evaluate the accuracy and robustness of the platform through actual tests.

## 2. Materials and Methods

### 2.1. Development of the Muscle Loading Platform

**Figure 1:**
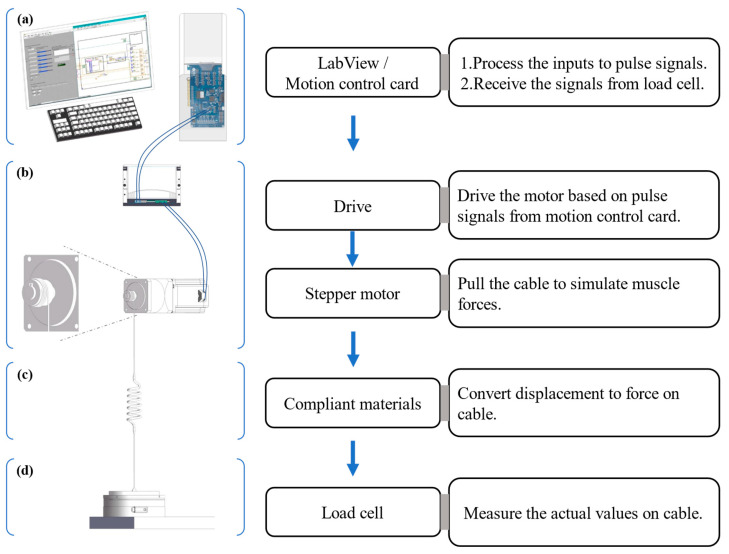
Overview of muscle force loading platform and the functions of main components. Four parts of the platform: (**a**) control system, (**b**) executive mechanism, (**c**) compliant materials and connecting cables, and (**d**) measuring equipment.

The hardware of the muscle loading platform was composed of four parts: the control system, executive device, compliant materials and connecting cables, and measuring equipment (Figure 1).

1. Control system. An industrial PC (ChengMing 3980, Dell Inc., Round Rock, TX, USA), into which a motion controller card (DMC3C00, Leadshine Co., Shenzhen, China) was inserted, was the main component of the control system. The PC was composed of an Intel Core i7-8700 CPU with 3.2 GHz~4.60 GHz, 8 Gb of RAM, and 512 Gb HDD. In addition, a connection board (ACC-XC00, Leadshine Co., China) bridged the PC and motor drive with dedicated cables.
2. Executive device. A stepper motor (86CM120, Leadshine Co., China) was driven by a motor drive (DMA882S, Leadshine Co., China) which worked at 20~80 V and 2.7~8.2 A, the latter powered by an adjustable power supplier (TDGC3-5000VA, Zhengxi Electric Technology Co., Ltd., Wenzhou, China). The 86CM120 was a two-phase stepper motor that had a step angle of 1.80 degrees and could deliver a holding torque of 12 N.m on a phase current of 6 A.
3. Compliant materials and connecting cables. Two groups of two kinds of industrial rubber bands (narrow band: 1.5 mm (thickness) × 20 mm (width) × 400 mm (1/2 length); wide band: 1.5 mm (thickness) × 30 mm (width) × 400 mm (1/2 length), Shands Inc., Shenzhen, China) played the compliant material role here. Each group contained six or eight rubber bands. A steel wire rope with a diameter of 1.5 mm connected the compliant material to the measuring equipment and the spool on the drive shaft of the stepper motor from the executive device (Figure 1).
4. Measuring equipment. A six degrees of freedom (6 DOF) force–torque transducer, the Omega 190 (ATI Industrial Automation, Inc., Apex, NC, USA) powered by a DC power regulator (LRS-50-24, MEAN WELL Co., Ltd., Taipei, China), was used as the load transducer with the sampling frequency of 100 Hz to measure the force generated in the cable.

A customized code was developed with LabView (V2018, National Instrument, Austin, TX, USA) on an industrial PC. The codes processed the inputs into pulse signals that could be recognized by the motor drive and sent the low-current signals to the motor drive through the motion control card. The motor drive powered by the power supplier took the low-current signals from the controller and amped them up into a high-current signal to correctly drive the stepper motor. Then, the simulated muscle force would be generated on the cable which connected the drive and compliant material. The forces were monitored through LabView codes by receiving mechanical signals from the load cell.

To verify the robustness of the test platform, we changed the compliant materials of the platform into two different hardware combinations to verify whether they can accurately reproduce the target muscle force values. (The first hardware combination was equipped with eight narrow bands, abbreviated as HDC1; the HDC2 group had six wide bands as compliant materials).

### 2.2. Numerical Computation Method to Reproduce Muscle Forces

#### 2.2.1. Obtaining the Target Muscle Forces

The magnitude and the changing pattern of muscle forces had unique characteristics. Here, we took the dynamic physiological muscle loading as the target values to evaluate the system’s ability to reproduce the muscle loads.

The knee was one of the most complex and frequently used joints of humans, with multiple muscles involved during functional activities. Here, we took the knee as our research object. Muscle forces were usually estimated in two fundamental ways—forward dynamics and inverse dynamics [30]. In fact, the patterns of muscle loading were similar whether they were predicted by the forward approach or inverse approach [30,31,32]. One [32] of the studies which estimated muscle forces using two pieces of software (AnyBody vers. 6.0 and OpenSim vers. 3.2) was randomly selected. GRABIT (MATLAB Central File Exchange, https://www.mathworks.com/matlabcentral/fileexchange/7173-grabit (accessed on 7 July 2022)), the MATLAB open-source toolkit designed to extract data points from coordinate-calibrated images [33,34,35], was used to grab data points in the figures from the published study we mentioned above. As a result, we obtained a total of 20 sets of data from 10 muscles (including 4 from quadriceps named rectus femoris, vastus lateralis, vastus medialis, and vastus intermedius; 4 from hamstrings, with 2 from biceps femoris, semitendinosus, and semimembranosus; and 2 belonging to calf muscles, the gastrocnemius lateralis and medialis), and the muscle forces were calculated during the gait cycle via inverse dynamics methods [32].

#### 2.2.2. Numerical Computation to Reproduce Muscle Forces

This part presents how to simulate the muscle forces through a numerical computation method. The muscle force—the percentage of the gait cycle obtained in the former chapter—is presented below:

(1) $$F_{e}=\varphi (e)$$

$F_{e}$: muscle force value at percentage *e* of the gait cycle;
φ: the relationship between force and time. The function *φ* represents a graphical correspondence between gait percentage and force value here.

The force–displacement relationship of compliant material could be obtained from material testing, as follows:

(2) $$G_{k}=h(k)$$

$G_{k}$: force on the material at the displacement of *k*;
*h*: the relationship between force and displacement. The function h represents a tabular correspondence between displacement and force.

Making use of Equations (1) and (2), and giving a gait cycle time *T*, we obtained the time–displacement curve, which represents muscle force reproduced by the compliant material. We cut the relationship curve into 1000 segments, and, for each segment, we used a cubic equation [36,37] to describe the relationship between time and displacement, as follows:

(3) $$i=f\left(t\right)=at^{3}+bt^{2}+ct+d$$

f: functional relationship of each segmental part of time *t* to displacement *i*;
*t*: time point;
*a*, *b*, *c*, *d*: unknown constants of a cubic function.

The four unknown constants *a*, *b*, *c*, *d* can be addressed by solving equations that consist of four sets of known points as input. Based on Equation (3), the derivative of *i* with respect to *t* could be expressed as follows, which presents the relationship between speed *v* and time *t*:

(4) $$v=\frac{di}{dt}=3at^{2}+2bt+c$$

*v*: cable speed at time *t*;
*a*, *b*, *c*: unknown constants of the cubic function.

For each muscle force curve, we obtained 1000 couples of motion instructs of the stepper motor with respect to speed *v* and time *t*. At each time point *t*, there are three corresponding kinematic parameters on the cable—speed, force, and power.

#### 2.2.3. Verification of Platform Performance

Any hardware has its upper limit of performance. When the motor drives the cable to generate muscle force, the platform may not meet the speed, force, or power requirements of the cable. Here, we will verify the performance of the platform to see whether it can meet the following testing requirements so as to ensure the safety of muscle reproducing tests.

The performance of the motor under the work condition that angular velocity changed linearly can be expressed as follows:

(5) $$T_{max}=J_{eq}\varepsilon =\frac{J_{eq}\cdot 2\pi n_{m}}{60t_{a}}$$

$T_{max}:$ maximum torque on the motor shaft, N·m;
$J_{eq}$: the motor moment of inertia, kg·m^2^;
ε: motor angular acceleration, rad/s^2^;
$n_{m}$: the speed of the motor shaft, r/min;
$t_{a}$: the time required for the motor to accelerate, s.

The speed *v* and the tensile force *F* on the steel cable in series with the compliant material were then derived as follows:

(6) $$F=\frac{T}{r_{s}}=\frac{J_{eq}\cdot 2\pi n_{m}}{60t_{a}}\cdot u\cdot \eta \cdot \frac{1}{\gamma }\cdot \frac{1}{r_{s}}$$

(7) $$v_{cable}=n_{m}\cdot \frac{1}{u}\cdot \pi r_{s}$$

u: gear ratio of the reducer, u = 5:1;
η: efficiency of motor and gear reducer, η = 0.80 [38];
γ: safety factor, γ = 1.2 [39];
$r_{s}$: radius of the spool on the motor shaft, $r_{s}$ = 1.43 × 10^−2^ m.

At each point *t*, the corresponding power *p* can be calculated as follows:

(8) $$p=F\cdot v_{cable}$$

*p*: real-time power on cable.

According to the previous ex vivo biomechanical studies [40,41,42,43,44], the presets of the testing duration (gait cycle time *T*) were set to 5 s, 10 s, 15 s, and 20 s. The three most challenging “extreme points” in the process of reproducing muscle force by elongating the compliant material, namely the maximum speed point, the maximum tensile force point, and the maximum power point, respectively, were considered to evaluate whether the performance of motor meets the speed, force and power requirements.

Under each hardware combination (HDC1 and HDC2), we evaluated whether the performance could meet the test requirements of 240 extreme points, which were derived from the curves of muscle forces that were calculated using two different pieces of software on 10 muscle groups, and each result was tested under four testing durations.

### 2.3. Muscle Force Reproducing Tests

Three groups of muscles (the quadriceps, the hamstring, and the calf muscles) surrounding the knee will be activated during walking or running. The forces from the same group exhibited a similar changing pattern; therefore, only one of them was randomly chosen to test here. Three muscles from different groups were selected, they were the gastrocnemius lateralis, the rectus femoris, and the semitendinosus. The changing pattern and magnitude of the three muscle loads are varied, and they are representative of a wide range of physiological loads. Each muscle contained two sets of data estimated using two pieces of software; thus, we brought in a total of six sets of data for testing. The test durations for HDC1 and HDC2 would be determined after performance verification. The shorter the test cycle, the greater the challenge for hardware. Therefore, the shortest test duration allowed by the hardware performance would be applied to complete the muscle forces reproducing tests for each hardware combination. To verify the robustness of the platform, each bundle of muscles was tested in eight cycles.

### 2.4. Statistical Analyses

Each set of actual muscle force measured using the force–torque transducer was filtered via the Butterworth filter and then averaged over the eight sets of data. A goodness-of-fit (GoF) test [36] was performed between the actual muscle force data set and the target data set. Each fit test would return a vector value (normalized mean squared error, NMSE) varying between −infinity and 1 (−∞ ~ 1). A fit value of 1 indicated it was no better than a straight line at matching expected data; 0 means perfect fit; −infinity signified a bad fit.

## 3. Results

### 3.1. Verification of Platform Performance

**Figure 2:**
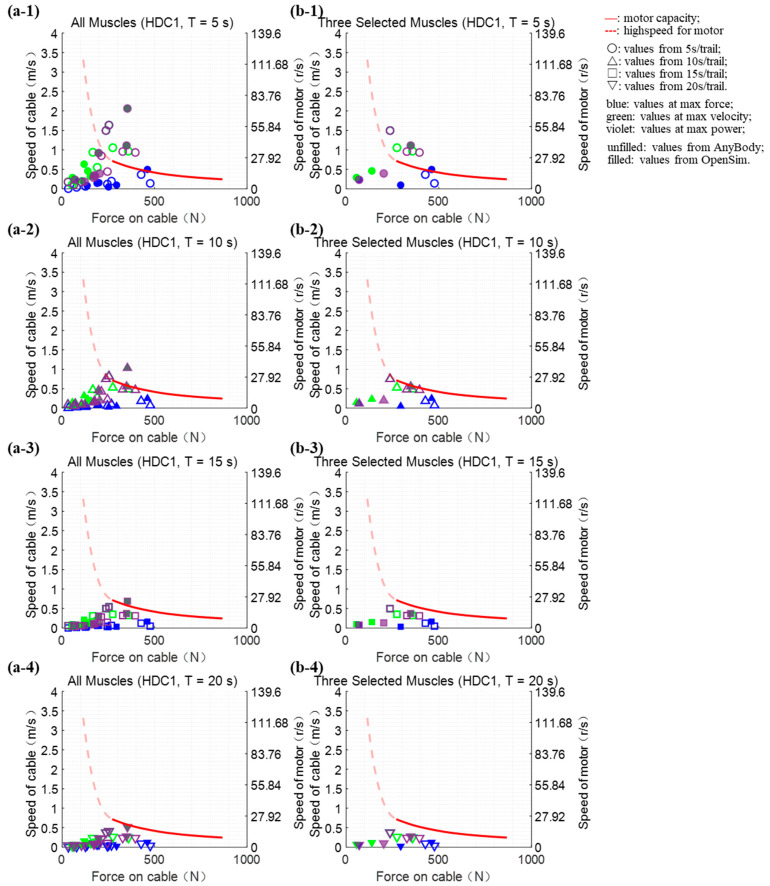
Verification of the platform performance under the HDC1. The platform performance is presented by the curves and the dashed part represents the motor at a high speed. Each dot indicates the “extreme point”, which refers to the maximum force or speed or power point. The dots below the curves mean the performance requirements of the corresponding “extreme point” could be satisfied. (**a1**–**a4**) all ten muscles (except gastrocnemius medialis because the “extreme points” of it were much beyond the capacity curve) evaluation schematic diagram; (**b1**–**b4**) three selected muscles (the gastrocnemius lateralis, the rectus femoris, and the semitendinosus) evaluation schematic diagram.

We carried out performance verification on 240 extreme points under HDC1 (Figure 2), which utilized six narrow industrial rubber bands in parallel as the compliant material. When the testing duration was set to 15 or 20 s, the force, speed, and power of all “extreme points” can be satisfied under HDC1. Just three “extreme points” exceeded the limit of the platform’s performance when the duration was 10 s, while when the test time was 5 s, the number was 11. When the evaluation was carried out on three selected muscles, the platform could meet the performance requirements of all “extreme points” when the test period was 10 s or longer. The following reproducing tests of three selected muscle forces would be run with a duration of 10 s under HDC1.

**Figure 3:**
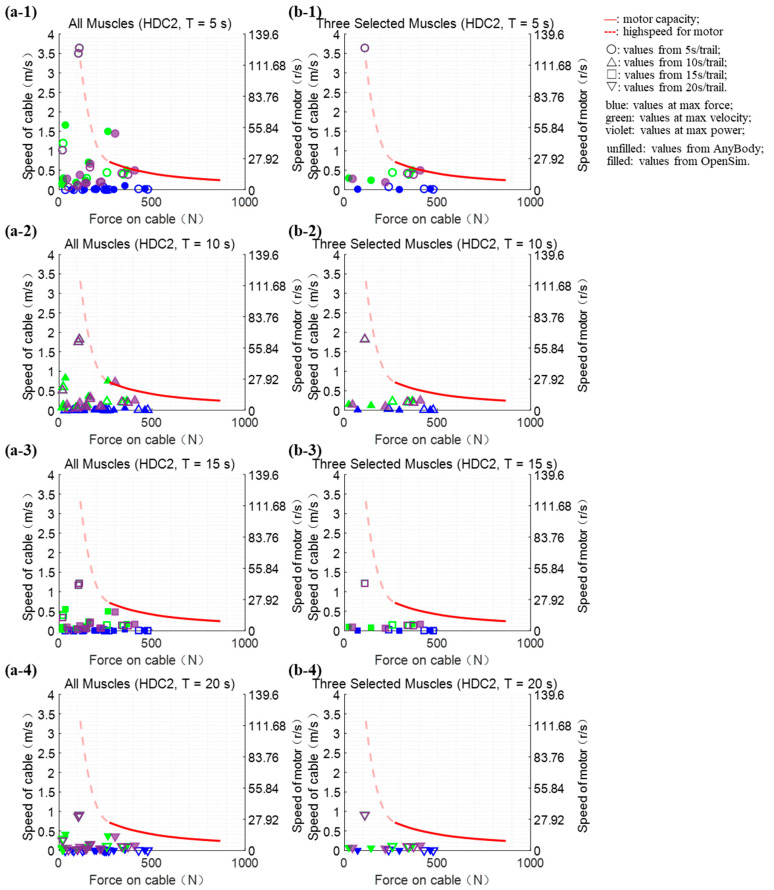
Verification of the platform performance under the HDC2. The platform performance is presented by the curves and the dashed part represents the motor at a high speed. Each dot indicates the “extreme point”, which refers to the maximum force or speed or power point. The dots below the curves mean the performance requirements of the corresponding “extreme point” could be satisfied. (**a1**–**a4**) all ten muscles (except gastrocnemius medialis because the “extreme points” of it were much beyond the capacity curve) evaluation schematic diagram; (**b1**–**b4**) three selected muscles (the gastrocnemius lateralis, the rectus femoris, and the semitendinosus) evaluation schematic diagram.

The performance verification on 240 extreme points was also evaluated under HDC2. Almost all “extreme points” of ten muscles (except the gastrocnemius medialis) were satisfied in the aspect of speed and force or power when the test cycle time was beyond 5 s, and only three “extreme points” from two muscles exceeded the performance required when the test duration was 5 s. All 72 “extreme points” from three selected muscles were subjected to the evaluation even though the test time was set to 5 s. The muscle force reproducing test ran at 5 s with the HDC2 (Figure 3).

### 3.2. Actual Muscle Forces Reproducing Tests

**Figure 4:**
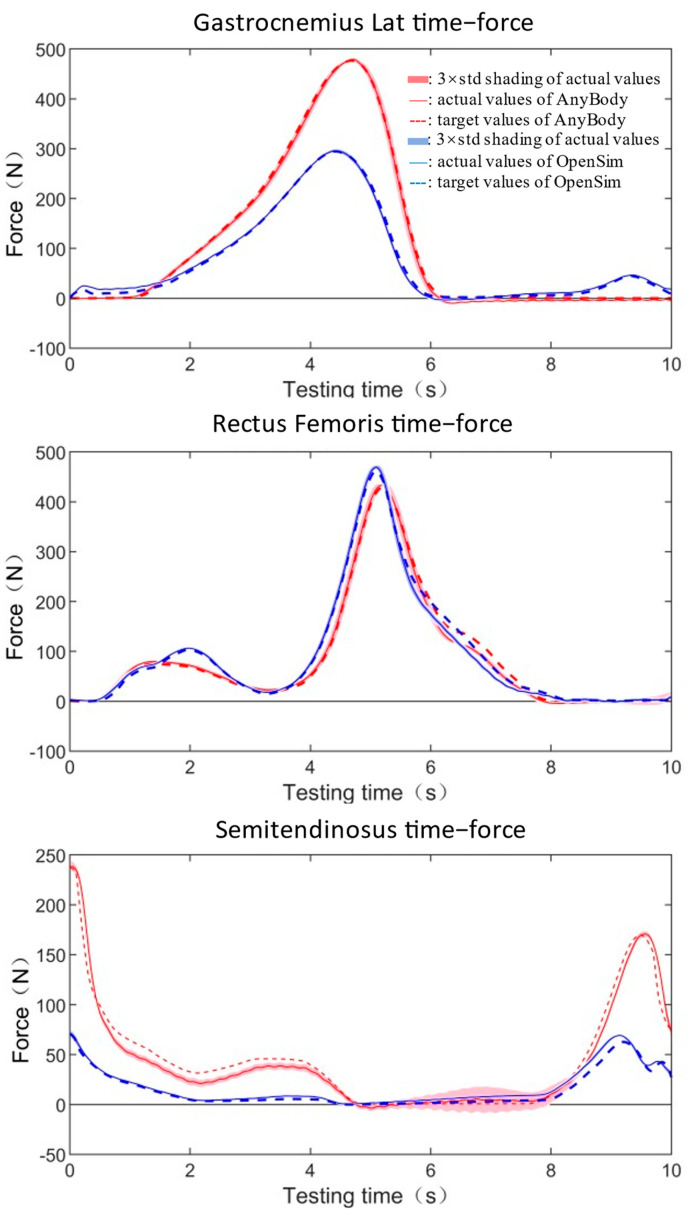
Muscle force reproducing results with HDC1 in 10 s. Dashed lines represent the target muscle force and the solid lines represent the actual force acquired using the measuring equipment from the testing system.

**Figure 5:**
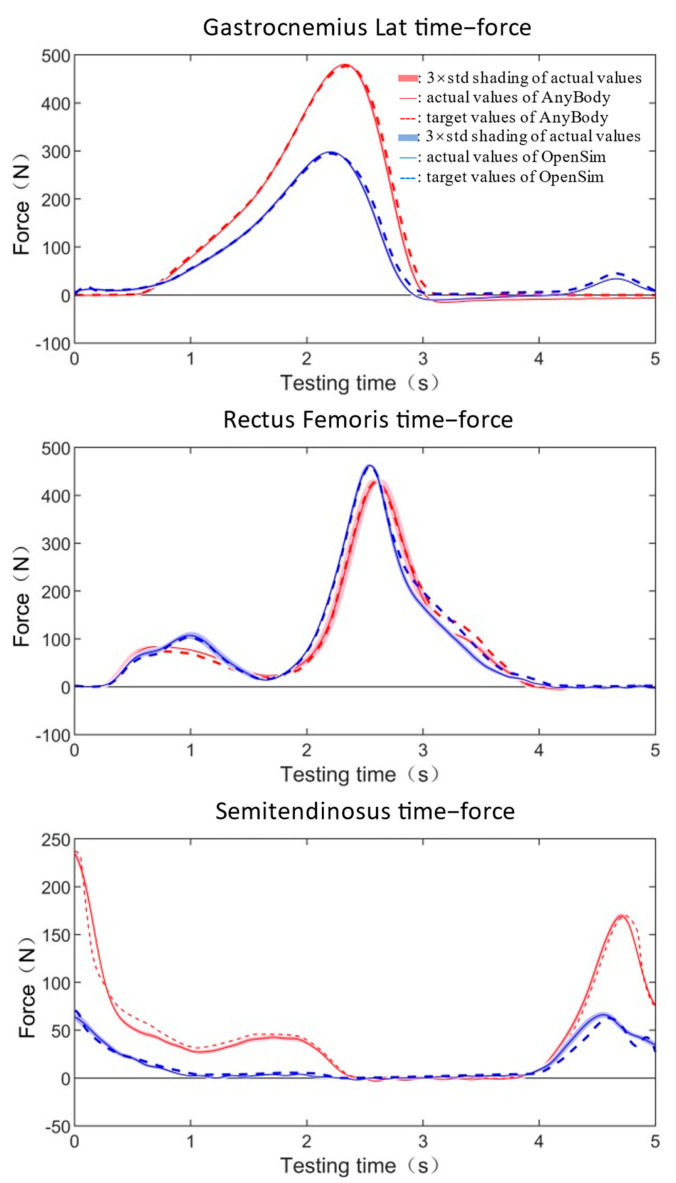
Muscle force reproducing results with HDC2 in 5 s. Dashed lines represent the target muscle force and solid lines represent the actual force acquired using the measuring equipment from the testing system.

The muscle forces reproduced by HDC1 and HDC2 showed a high degree of consistency between the target force values and the actual measured force values (Table 1 and Table 2, Figure 4 and Figure 5). Under the condition of HDC1, all muscle forces were reproducing in 10 s with almost no time delay (Figure 4). The mean standard deviations of repeat tests for the entire test period were between 0.34 N and 2.79 N. The errors between actual maximum force and target maximum force were in a range of 0.53~1.81%. The GoF factors were all below 0.06 (0.00~0.06), which was close to a perfect fit (Table 1).

Similarly, nearly no time delays were observed (Figure 5) in the HDC2 group, which finished all tests in 5 s with errors of maximum force in a range of −6.20%~1.42% (Table 2). The small mean standard deviations (0.68 N~2.93 N) indicated the platform with the condition of HDC2 was still robust. The approximate perfect fit behaviors were observed for all fitting results (0.00~0.05) (Table 2).

## 4. Discussion

This study established a dynamic physiological muscle force reproduction platform. The accuracy and robustness of the platform were validated with actual tests. This platform was built to produce muscle forces during functional activities, mainly manifested as the accuracy of muscle strength (errors: −6.2%~1.81%) and the consistency of changing patterns (GoF factors in a range of 0.00~0.06). In addition, the system exhibits a high order of robustness, as eight testing trials for each muscle force all maintained a considerably low mean standard deviation level regardless of whether the hardware combination was changed (standard ranges of HDC1: 0.34 N~2.79 N vs. HD2: 0.68 N~2.93 N).

As the “drive” of joints in vivo, muscle forces are the prerequisite for establishing a bionic environment in vitro. Some studies attempted to simulate muscle forces in in vitro mechanical experiments through various methods. Panjabi et al. added a set of static muscle forces to the spinal units which were driven by a pure torque and found that the muscle forces increased the flexion range of motion by as much as 1.5 times [22]. Li et al. applied the same approach to the knee joint and found that adding the quadriceps and hamstrings at 30° of knee flexion resulted in a 66.0% increase in tibial anterior translation and a 78.7% increase in internal rotation compared to pure moment loading [23]. Clearly, muscle forces have a significant impact on kinematics and kinetics. However, these forces were a set of static forces that did not match the muscle loading in vivo. Ferreira et al. utilized dynamic muscle loading to drive a forearm to complete an active flexion [28]. However, the muscle forces were not quantitative inputs, as neither the reproducibility of physiological loading nor their accuracy can be evaluated. Recently, Schall et al. employed dynamic muscle loads calculated via inverse dynamics to the knee specimens to complete the jumping movement, but the results showed that the “prime mover” quadriceps muscle forces were 10~15% lower and there was a time delay of 9.5~14.3% even though the preload of muscle forces started at about 45% of the maximum force value [29]. All these studies failed to accurately reproduce the dynamic muscle forces in vitro, resulting in the differences in kinematics and/or mechanical environment between specimens in vitro and joints in vivo, which is one of the reasons that they are restrictive to solving some partial problems. The platform we established could generate dynamic muscle forces with an error of less than −6.2% (a value of 4.36 N) and no time delay in reaching the maximum value.

The magnitude and timing alignment of simulated muscle forces are very important for building a musculoskeletal experiment in vitro. The size and bearing capacity of each joint of the human body is different, and the magnitude and changing pattern of muscle loading are also different. It is very meaningful that a platform can provide great tolerance to accommodate more muscles. In previous studies, the muscles forces applied cannot reach the magnitude of the in situ forces even if they are static forces; for example, the sum of quadriceps forces is about 120 N~260 N [45,46,47,48], which is not comparable to the force in vivo. The current platform can achieve the output with the maximum force of nearly 500 N in 5 s and ensure the accuracy of the measured values is as high as when the muscle force is small. Timing alignment is a critical issue for the reconstruction of the joint mechanical environment besides muscle force. The movement of joints relies on the cooperation of multiple groups of muscles, which requires different muscle forces to be completely consistent in time sequence, or the joints will suffer from the unbalanced force, which will damage the samples or destabilize the joints. Schall et al. loaded the dynamic muscle forces into the knee joint musculoskeletal experiment, but the time for the muscle forces to reach the maximum value was about 9.5~14.3% slower [29]. The test results prove that the current platform has a fast dynamic response capability, no time delay is observed in the current test in either the time for the target force to reach the maximum value or between two extreme values.

The current study proposes a practical muscle force bionic platform, which can be flexibly transplanted to the in vitro biomechanical experimental platform. Some studies apply industrial pneumatic [49] or hydraulic [50] cylinders directly to tendons with a rigid cable to simulate muscle force; they can generate adequate forces but their motion accuracy is not high [51], easily resulting in large unexpected forces generated by rigid connections or direct cable breakage. In the current system, a stepper motor with a stepping angle of 1.8° is used as the actuator, and compliant material that mimics the structure of the ligaments in vivo is attached to the connecting cable to ensure the accuracy of forces on the cable and buffer the motion error. Admittedly, stepper motors usually cannot output substantial force like pneumatic or hydraulic devices; however, the current platform successfully increases torque/forces and ensures the short duration of the test cycle by employing a reducer. The reducer increases the motor revolutions, which makes the testing duration increase relatively. However, too fast a testing speed for in vitro specimens would also trigger the viscous effect of devitalized soft tissues (like joint capsules, and patellar fat pads, among others) [40] or amplify the effect of mechanical inertia [41], which brought unpredictable force to the specimen. Cycle periods of 4~20 s or longer are the commonly applied length of in vitro experiments, whether it is in the shoulder [42], hip [43], knee [40], and ankle [44] joint tests or in spinal [41] experiments. In general, the hardware combination of the current platform is simple and stable, and it can accomplish the reproduction of muscle loading with a short duration.

This study has three main limitations. Firstly, we only tested one muscle loading at a time and did not test the coordination of multiple groups of muscles. In the next study, we will evaluate the cooperative effect of multiple roads of muscle loading on cadaver samples. Because each muscle is produced by a separate muscle loading system, it can be predicted that there will be no interference between them. Secondly, only the compliant materials were changed in the hardware combinations to evaluate the robustness of the platform, while other hardware remained unchanged. It can be seen from the results that the change in hardware parameters will only affect the length of time to produce the muscle loading and will not affect the accuracy and changing pattern of forces. Finally, we only utilized materials made of rubber as compliant materials for the current study; thus, the results might not be as good as ours when the compliant material is replaced with other materials such as a viscoelastic material. Because the goal of this study is to generate dynamic muscle forces at the attachment point of the specimen, we advocate the use of materials with better mechanical properties as compliant materials to simplify the computation and experimental processes.

## 5. Conclusions

A novel platform and corresponding numerical computation method to reproduce physiological muscle forces are established in the current study. The validation results show that the platform has high accuracy and can reproduce the target muscle force with a low error and a high goodness-of-fit coefficient from 0 to 0.06 (close to a perfect fit). The platform also shows high robustness. Repeated tests maintain a small average deviation (0.34 N~2.93 N), and the muscle loading can still be reproduced with high accuracy after changing the hardware combination. The current stable muscle loading platform has great potential to be applied in a future musculoskeletal loading system.

## Figures and Tables

**Table 1:** Characteristics of muscle force reproducing tests under HDC1 with a cycle time of 10 s.

		Maximum Force			Mean Standard	GoF (NMSE)
		Target Value	Actual Value	Error (%)		
gastrocnemius lateralis	AnyBody	476.57	479.10	0.53	2.23	0.00
	OpenSim	294.45	296.03	0.54	0.87	0.00
rectus femoris	AnyBody	427.68	433.11	1.27	2.79	0.01
	OpenSim	461.52	468.95	1.61	2.02	0.01
semitendinosus	AnyBody	237.00	238.50	0.63	1.92	0.03
	OpenSim	70.36	71.63	1.81	0.34	0.06

**Table 2:** Characteristics of muscle force reproducing tests under HDC2 with a cycle time of 5 s.

		Maximum Force			Mean Standard	GoF (NMSE)
		Target Value	Actual Value	Error (%)		
gastrocnemius lateralis	AnyBody	476.57	480.02	0.72	1.11	0.00
	OpenSim	294.45	298.62	1.42	1.13	0.00
rectus femoris	AnyBody	427.68	427.63	−0.01	2.93	0.01
	OpenSim	461.52	464.55	0.66	2.21	0.01
semitendinosus	AnyBody	237.00	234.19	−1.19	0.92	0.02
	OpenSim	70.36	66.00	−6.20	0.68	0.05

## Data Availability

All data have been included in the manuscript.
